# Feasibility of anchoring active fixation quadripolar lead to the main body of the dilated coronary sinus in a patient with persistent left superior vena cava: a case report

**DOI:** 10.1093/ehjcr/ytaf008

**Published:** 2025-01-16

**Authors:** Yuka Taguchi, Junya Hosoda, Akira Horigome, Toshiyuki Ishikawa

**Affiliations:** Department of Cardiology, Yokohama City University Hospital, 3–9 Fukuura, Kanazawa-Ku, Yokohama city, Kanagawa 236-0004, Japan; Department of Cardiology, Yokohama City University Hospital, 3–9 Fukuura, Kanazawa-Ku, Yokohama city, Kanagawa 236-0004, Japan; Department of Cardiology, Yokohama City University Hospital, 3–9 Fukuura, Kanazawa-Ku, Yokohama city, Kanagawa 236-0004, Japan; Department of Cardiology, Yokohama City University Hospital, 3–9 Fukuura, Kanazawa-Ku, Yokohama city, Kanagawa 236-0004, Japan

**Keywords:** Active fixation lead, Biventricular pacing, Pacing-induced cardiomyopathy, Lead extraction, Attain stability, Case report

## Abstract

**Background:**

Coronary sinus (CS) lead placement in persistent left superior vena cava (PLSVC) cases is challenging because of the poor backup force of the guiding catheter within the enlarged CS. Active fixation Quadripolar leads (Attain Stability™ Quad 4798, Medtronic) can expand choice to CS branches with limited access; however, no cases of anchoring to the main body of the CS have been published to date.

**Case summary:**

We describe a case of cardiac resynchronization therapy pacemaker upgrade in a 79-year-old female who developed pacing-induced cardiomyopathy after pacemaker implantation via the right superior vena cava (SVC) for atrioventricular block eight years ago wherein PLSVC was revealed during the procedure. Retrograde giant CS angiography via SVC confirmed the lateral vein ostium. Attain Stability Quadripolar lead was selected; however, due to the tortuousness and stenosis of the target vein, the proximal electrodes could not advance into the target vein. Therefore, the side helix between the third and fourth electrodes was crimped to the anterior wall of the giant CS using the distal end curve of the subselection catheter and successfully screwed into the main body of the CS. At more than 6 months, left ventricular ejection fraction improved without lead dislodgement.

**Discussion:**

Fixation of CS lead to the main body of the dilated CS was feasible by devising a guiding catheter and a subselection catheter. Nevertheless, the safety of active fixation lead retraction after long-term indwelling in CS is unknown and it should be carefully considered.

**Learning points**

- If the proximal electrodes cannot advance into the coronary sinus branch, the risk of dislodgement is high with a passive fixation lead; however, in those cases where coronary sinus branches are limited, the active fixation Attain Stability Quadripolar lead provides a wider choice of retention sites, and fixation to the main body of the coronary sinus is feasible.
- The distal end curve of the subselection catheter and guiding catheter were useful to crimp the side helix to the vessel wall to fix the dilated coronary sinus associated with the persistent left superior vena cava.

## Introduction

Coronary sinus (CS) lead placement in patients with persistent left superior vena cava (PLSVC) is still challenging due to complex anatomy.^1^ In this report, we described a case of a cardiac resynchronization therapy pacemaker (CRT-P) upgrade who has both a right superior vena cava (SVC) and PLSVC without an innominate vein (Type III B),^2^ in which CS lead was fixed to the main body of the dilated CS using an active fixation quadripolar lead equipped with a side helix.

## Summary figure

**Figure:**
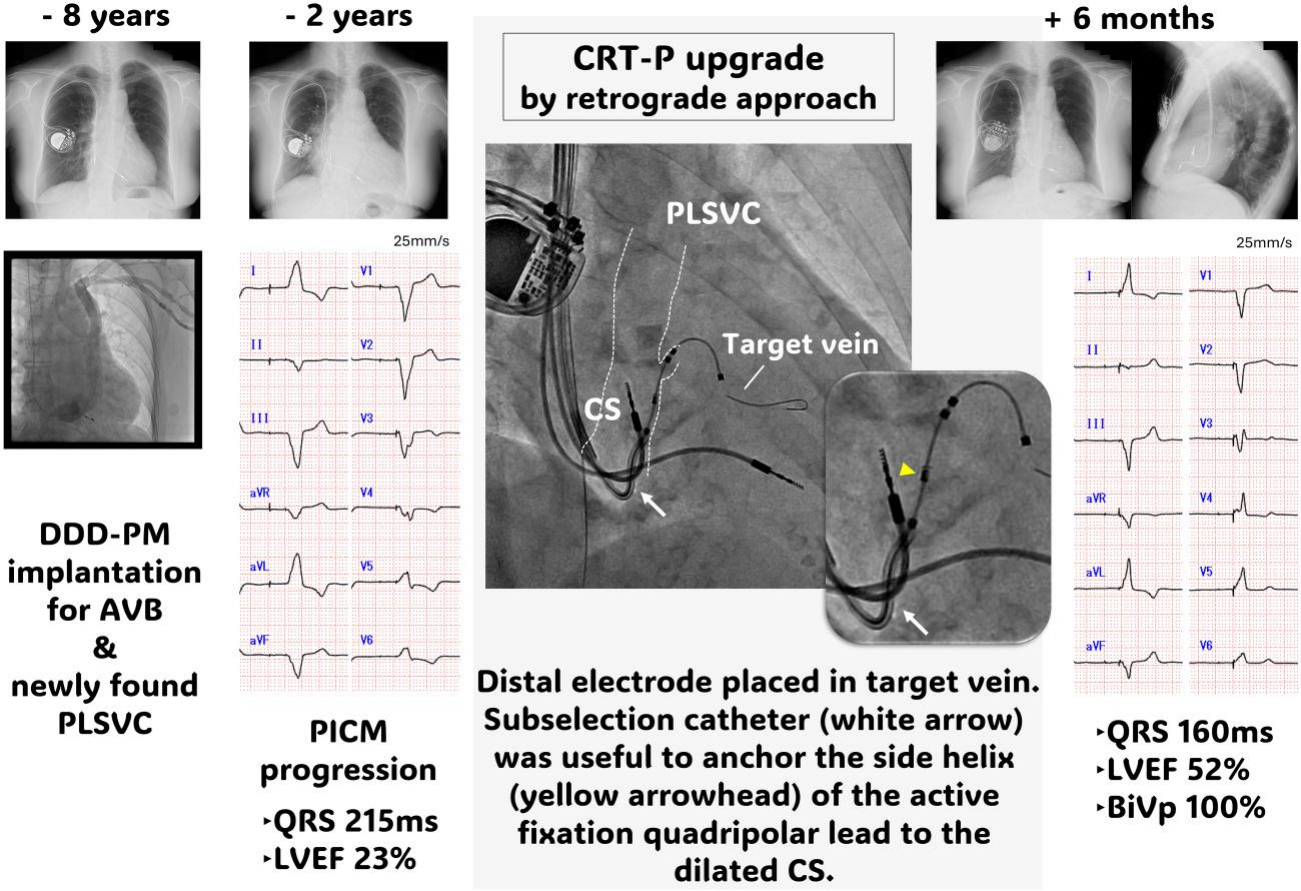
AP, anteroposterior; AVB, atrioventricular block; BiVp, biventricular pacing; CRT-P, ardiac resynchronization therapy pacemaker; CS, coronary sinus; LVEF, left ventricular ejection fraction; PICM, pacing-induced cardiomyopathy; PLSVC, persistent left superior vena cava; PM, pacemaker; RAO, right anterior oblique.

## Case presentation

**Figure 1:**
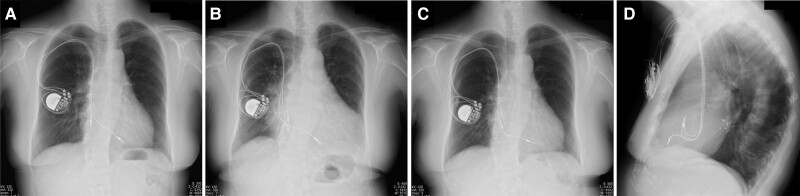
Chest X-ray images after pacemaker implantation (*A*), when heart failure developed 6 years later due to pacing-induced cardiomyopathy (*B*), and 6 months after cardiac resynchronization therapy pacemaker upgrade in frontal view (*C*) and lateral view (*D*). CS, coronary sinus; CRT-P, cardiac resynchronization therapy pacemaker; PLSVC, persistent left superior vena cava.

**Figure 2:**
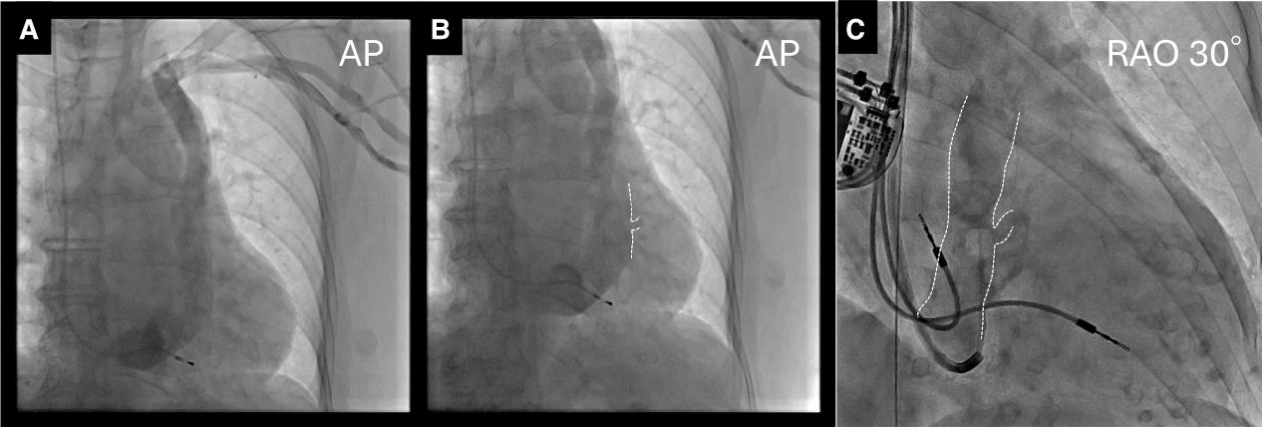
(*A* and *B*) Venography before pacemaker implantation revealed a persistent left superior vena cava without an innominate vein and a faint contrast of the lateral venous ostium branching from the coronary sinus (dotted line). A temporary right ventricular lead was inserted via the right superior vena cava. (*C*) Retrograde coronary sinus venography during cardiac resynchronization therapy upgrade delineates the contours of the giant coronary sinus, persistent left superior vena cava, and the lateral venous ostium region (dotted line). AP, anteroposterior; CS, coronary sinus; CRT-P, cardiac resynchronization therapy pacemaker; PLSVC, persistent left superior vena cava.

A 79-year-old Japanese female underwent transvenous lead pacemaker implantation for complete atrioventricular block with normal left ventricular ejection function (LVEF) (*Figure 1A*) and venography during the procedure revealed PLSVC draining into dilated CS and right SVC without an innominate vein 8 years previously. Therefore, the pacemaker was implanted on the right side via the right SVC. Six years later, the patient developed dyspnoea on exertion and presented with eyelid and pedal oedema. Blood pressure was 137/89 mmHg and oxygen saturation was 98% on room air. The right ventricular (RV) pacing rate was consistently 100% in device check, but the QRS duration was prolonged from 170 to 215 ms after initial implantation. Chest X-ray showed cardiac enlargement and pleural effusion (*Figure 1B*), echocardiography a severely deteriorated LVEF of 23% and desynchrony due to RV pacing. B-type natriuretic peptide level was elevated at 1287 pg/mL but high-sensitivity troponin levels were normal. By ruling out ischaemic heart disease and other cardiomyopathies on close inspection, the clinical course indicated pacing-induced cardiomyopathy. Although an upgrade to CRT-P was recommended, given the uncertainty that CS lead placement could be implanted via PLSVC, we preferred optimal medical therapy for heart failure that included diuretics, a mineralocorticoid receptor antagonist, an angiotensin receptor/neprilysin inhibitor, a beta blocker, and a sodium glucose co-transporter 2 inhibitor according to the current guidelines. These medications resulted in improved symptoms and an LVEF of 41%. During the following 2 years, no further improvement in EF, newly developed noise episodes in the atrial lead, and generator battery wastage were confirmed. A review of venography at the time of pacemaker implantation identified a lateral vein ostium branching from the dilated CS (*Figure 2A* and *B*, Supplementary material online, *Videos S1*). Cardiac resynchronization therapy pacemaker upgrade and additional atrial lead placement were attempted with patient consent.

**Figure 3:**
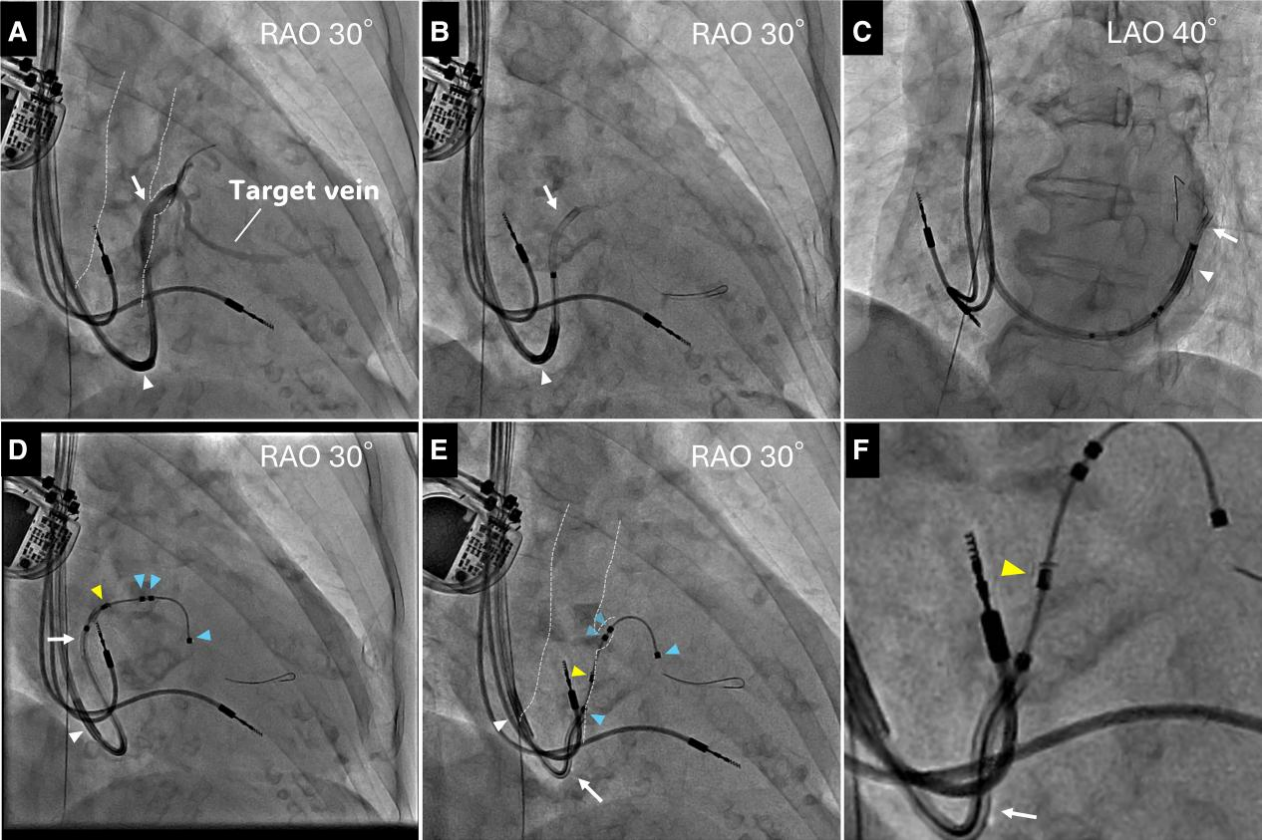
A subselection catheter (white arrow) was cannulated into the lateral vein and backed up with a guiding catheter (white arrowhead). A contrast from the catheter showed that the target vein was steeply bent and stenotic at the end of the bend (*A*). The guidewire crossed the flexure and deep into the lateral vein in right anterior oblique (*B*), and left anterior oblique view (*C*). Attain Stability Quadripolar lead (blue arrowheads) was delivered into the lateral vein over the wire but could not pass over the bent stenosis, and the subselection catheter was pushed out when the lead was attempted to advance (*D*). A subselection catheter was pulled to expose the third and fourth electrodes, and the side helix (yellow arrowhead) between them was pressurized against the anterior wall of the dilated coronary sinus using the distal end curve of the catheter (*E*). The lead was successfully anchored to this location by clockwise rotating the lead body. Enlarged image of E (*F*). CS, coronary sinus; LAO, left anterior oblique; RAO, right anterior oblique.

Retrograde venography using an angiographic balloon catheter through a guiding catheter (Attain Command™+SureValve™ 6250VIC; Medtronic plc, Dublin, Ireland) revealed an enlarged CS with a slightly contrasting lateral vein ostium at the anterior wall (*Figure 2C*, Supplementary material online, *Videos S2*), which was identified as the target vein. No other branches were visible. Selective insertion into the lateral vein using a subselection catheter (Attain Select™ II + SureValve™ 6248VI-90, Medtronic) inside the guiding catheter was achieved, and contrast infusion revealed that the lateral vein was steeply tortuous (*Figure 3A*). Because of the giant CS, the risk of lead dislodgement was considered high; thus, an active fixation quadripolar lead (Attain Stability™ Quad 4798, Medtronic) was selected and delivered over the guidewire (*Figure 3B* and *C*). The distal electrode crossed a strong flexure of the target vein but did not pass the subsequent stenosis (*Figure 3D*); thus, the proximal electrodes could not advance into the target vein and remained within the CS. Because the pacing threshold of the distal electrode was acceptable at 1.5 V/0.4 ms and other branches were difficult to perceive, we attempted helix lead fixation to the main body of the enlarged CS. The subselection catheter was pulled into the guiding catheter to expose the third and fourth electrodes and the side helix between them, and the helix was pressurized against the anterior wall using the distal end curve of the subselection catheter (*Figure 3E* and *F*). The lead body was then turned clockwise in 10 rotations and pulled to confirm successful fixation. Additional atrial lead placement was subsequently performed. QRS duration was shortened to 160 ms after CRT pacing. At 6 months of follow-up, biventricular pacing rate was 100%, and LVEF improved to 52%. No lead dislodgement was observed on chest X-ray (*Figure 1C* and *D*), and the lead parameters remained within acceptable limits.

## Discussion

PLSVC is the most common congenital anomaly of venous return, with a prevalence of approximately 0.5% in the general population.^3^ Most patients are asymptomatic, incidentally identified by echocardiography or other imaging modalities, or often recognized on venography at the time of cardiac device implantation.^3,4^

Several case reports of CS lead placement in these populations using active fixation lead have been published.^1,5–8^ The success of these procedures is highly dependent on individual concomitant vascular anomalies; isolated PLSVC without SVC (Type II), coexistent SVC with or without innominate vein (Type IIIa/b), presence of CS atresia, location of CS branches, and target vein diameters and flexures. Based on these factors, placement of a CV lead in these populations is challenging even with preoperative imaging.

To the extent of our knowledge, this is the first report of a side helix lead anchored to the main body of an enlarged CS associated with PLSVC. The procedure was feasible and showed clinical benefits without lead dislodgement. In most cases, CV leads were placed via the PLSVC. In an enlarged CS, it is generally difficult to stabilize the lead because of the poor backup force of the guiding sheath. Attain Stability Quadripolar lead is designed with a side helix between the third and fourth electrodes, which is coiled into the vessel wall by lead body rotation^9^ but cannot be fixed unless the helix is crimped to the vessel wall. In this case, however, the guiding catheter and distal end curve of the subselection catheter via the right SVC allowed stable crimping of the proximal electrodes and the side helix to the anterior wall of the CS during lead rotation and coiling of the side helix into the CS. This is a notable key to the success of this procedure.

Although conduction system pacing (CSP) is an alternative therapy, CRT was selected as the first choice for two reasons. First, the patient presented with heart failure with severely reduced ejection fraction. Cardiac resynchronization therapy is well established with abundant evidence in such cases, whereas the long-term outcomes are not clear for CSP to date. Second, there is a concern that the placement of a second RV lead in addition to the pre-existing RV lead may contribute to tricuspid regurgitation. Referring to the available guidelines,^10^ CSP was considered when CRT was technically infeasible or the patient was a CRT non-responder. If CSP cannot also be achieved, surgical left ventricular lead placement should be considered.

Although the Attain Stability™ Quadripolar lead has been reported as a retractable lead compared with the predecessor active fixation lead Star fix™ (Medtronic),^11,12^ the extraction outcomes after long-term dwelling are unknown. A recent multicenter retrospective report demonstrated a high extraction success rate (92% success and 8% partial success) and no procedure-related complications in 26 active fixation CS leads (mean duration of implantation 2 ± 1.6 years); however, 35% of the patients had to use extraction sheaths due to tissue adhesions within the CS.^13^ Injury to giant CS may carry a higher risk of more serious complications than injury to CS branches. Although this procedure should be well considered, the Attain Stability Quadripolar lead could provide more retention sites in cases with limited accessible veins.

## Lead author biography


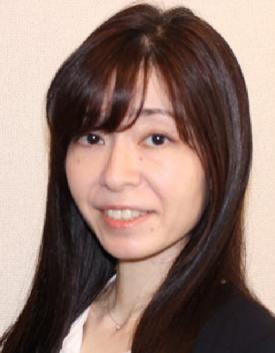


Dr Yuka Taguchi graduated from Hamamatsu University School of Medicine in 2009 and received her doctorate in Medical Science from Yokohama City University in 2021. In recent years, She has been working at Yokohama City University Hospital, specializing in the field of arrhythmia, as a board-certified member of the Japanese Heart Rhythm Society.

## Supplementary Material

ytaf008_Supplementary_Data

## Data Availability

The data underlying this article will be shared on reasonable request to the corresponding author.

## Notes

**Consent:** The authors confirm written consent for the submission and publication of this case report including images. The written consent form was compliant with COPE guidelines.
